# NK Cells Under Hypoxia: The Two Faces of Vascularization in Tumor and Pregnancy

**DOI:** 10.3389/fimmu.2022.924775

**Published:** 2022-06-13

**Authors:** Irene Garcés-Lázaro, Rebecca Kotzur, Adelheid Cerwenka, Ofer Mandelboim

**Affiliations:** ^1^ Department of Immunobiochemistry, Mannheim Institute of Innate Immunosciences (MI3), Medical Faculty Mannheim, Heidelberg University, Mannheim, Germany; ^2^ The Lautenberg Center for General and Tumor Immunology, Institute for Medical Research Israel-Canada, The Hebrew University Hadassah Medical School, Jerusalem, Israel; ^3^ European Center for Angioscience (ECAS), Medical Faculty Mannheim, Heidelberg University, Mannheim, Germany

**Keywords:** hypoxia, NK cells, vascularization, tumor microenvironment, pregnancy

## Abstract

Environmental conditions greatly shape the phenotype and function of immune cells. Specifically, hypoxic conditions that exist within tissues and organs have been reported to affect both the adaptive and the innate immune system. Natural killer (NK) cells belong to the innate immune system. They are among the first immune cells responding to infections and are involved in tumor surveillance. NK cells produce cytokines that shape other innate and adaptive immune cells, and they produce cytolytic molecules leading to target cell killing. Therefore, they are not only involved in steady state tissue homeostasis, but also in pathogen and tumor clearance. Hence, understanding the role of NK cells in pathological and physiological immune biology is an emerging field. To date, it remains incompletely understood how the tissue microenvironment shapes NK cell phenotype and function. In particular, the impact of low oxygen concentrations in tissues on NK cell reactivity has not been systematically dissected. Here, we present a comprehensive review focusing on two highly compelling hypoxic tissue environments, the tumor microenvironment (pathological) and the decidua (physiological) and compare their impact on NK cell reactivity.

## 1 Introduction

### 1.1 Definition of Hypoxia

Oxygen concentration is heterogeneous depending on the tissue contexture. The atmospheric O_2_ partial pressure is 159 mmHg, equivalent to 21% from total air fraction. This oxygen concentration is considered as “standard”, defined as normoxia (1). The atmospheric air is inhaled through the respiratory system and is exchanged in the alveolar-capillary barrier into the pulmonary veins. Pulmonary veins transport oxygen to the heart, where it is collected by the arteries. The arteries contain a partial pressure of 75 – 100 mmHg (depending on the altitude), and gradually reduce their size to the tissue arterioles and capillary vessels. Along the tissue irrigation by the capillary net, oxygen is diffused due to the gradient of pressure namely the difference in pressures between the transporter cell and the cytosol of the receptor cell. The quantity and speed of oxygen diffusion depends on barrier thickness, surface of area of diffusion and the metabolic request from surrounding tissues. In order to distribute the oxygen, the hemoglobin inside red blood cells in lungs binds to the oxygen forming oxyhemoglobin. Hemoglobin is activated by high CO_2_ concentrations, which triggers its high avidity for oxygen in its *taut* form (tense) and releases oxygen. On the other hand, the relaxed form is activated, when CO_2_ concentration is low, capturing O_2_ (2). Therefore, hemoglobin controls the gas exchange between vessels and tissues, regulated by CO_2_ concentration and O_2_ demand in the different compartments. Thus, the circuit from pulmonary veins to pulmonary arteries contains a distribution net with arterioles, venules and capillaries, which supply the tissues with O_2_ and nutrients (2).

Because each tissue requires different O_2_ concentrations, each compartment has its own oxygenation concentration considered as healthy; known as “tissue normoxia” or physioxia (2). For example, arterial blood contains 13% O2, venous blood has ≈ 5% O2 and cell culture conditions are often set to 19.95% O_2_ (2). On the other hand, when the oxygen concentration is lower than physioxia, it is defined as hypoxia (lack of oxygenation) (3). Hypoxia is highly relevant for physiology and disease, affecting many different functions, including cell metabolism and tissue structure with impact on therapeutic approaches (4).

### 1.2 Impact of Hypoxia in Tissues

Hypoxia is a regulator of multiple aspects of cell biology, including cell metabolism, cell cycle, angiogenesis, erythropoiesis and inflammation (5). It regulates the tricarboxylic acid cycle (TCA) in mitochondria and is involved in the Warburg effect. This effect is a phenomenon, which occurs when cells display high rates of glucose uptake by aerobic glycolysis. Under hypoxia, the transcription factors, hypoxia-inducible factors (HIFs) (6, 7), increase transcription of enzymes involved in cell metabolism including the kinase/phosphatase family.

In addition, HIF promoters target p27 and p21 resulting in higher transcription rates. Both proteins are direct inhibitors of CDK2, cyclin E/A and p53, enhancing cell cycle arrest (8). Most importantly, hypoxia affects angiogenesis and inflammation, which is highly relevant for tissue architecture.

#### 1.2.1 Angiogenesis

Under hypoxia, angiogenesis and erythropoiesis are enhanced by inducing the transcription of the vascular endothelial factor (VEGF), platelet-derived growth factor-β (PDGF-β), increasing iron transport by transferrins and inducing the production of erythropoietin (EPO) (5, 9, 10). In context of disease, EPO has been used for therapy for highly vascularized tissues, such as the kidney in chronic kidney disease (CKD). In these studies, HIFs increased EPO in serum and reduced the mortality risk (11). In addition, it has been recently shown that there is an epigenetic mechanism behind the hypoxia-dependent neoangiogenesis in breast cancer and hepatocellular carcinoma cells. Under hypoxia, HIF-1α activates transcription of the peptidylarginine deiminase 4 (PADI4), which regulates the histone access in the hypoxia response elements (HREs). Accordingly, VEGF and EPO are upregulated, resulting in angiogenesis and supporting tumor growth (12). Overall, there is clear evidence that under hypoxia, there is an increase in vascularization by the promotion of several angiogenic genes such as EPO or VEGF. Depending on the tissue, hypoxic angiogenesis may be beneficial (CKD), or deleterious (tumor growth).

#### 1.2.2 Inflammation

Hypoxia is also known to stimulate inflammation, measured by high IL-6 production and C-reactive protein in subjects exposed to high-altitude hypoxia (13). In addition to environmental hypoxia, tissue ischemia is reported to induce graft *vs* host disease in kidney transplants (14). The tissue rejection was linked to high toll-like receptor 4 activity in the transplanted kidney after ischemia, which induced an inflammatory response that correlated with high TNF-α production and low graft function. Moreover, the hypoxic niche directly affects both innate immune cells including NK or γδT cells, and adaptive immune cells such as T cells (15–18). In this review, we will focus on the role of NK cells. NK cells exposed to hypoxic conditions showed an increase in the expression of genes involved in glycolysis, gluconeogenesis and glucose transport, non-glycolytic metabolism, ion transport, apoptosis, stress response, proliferation, transcription and signaling activities (18). In addition, the evaluation of chemokines and cytokines secretion under hypoxia revealed its impact on NK migratory and chemotactic capability, including their infiltration into tumors. Accordingly, hypoxia-exposed NK cells showed an increase in the expression of genes relevant for angiogenesis, apoptosis inhibition, tumor progression and immunosuppression (18). Taken together, these data suggest that hypoxia is able to regulate NK cell immune responses (18). Moreover, NK cell cytotoxicity was significantly decreased under hypoxic conditions associated with a reduction of secretion of lytic agents and receptor/ligand interactions (19). In contrast, the antibody dependent cellular cytotoxicity (ADCC), a powerful triggering of NK cell activity activated by the Fc region of IgG antibodies, was not altered by hypoxia highly relevant for the use of therapeutic antibodies as therapy for solid tumors (20).

### 1.3 Hypoxia Inducible Factors

The cellular mechanisms triggered by the lack of oxygen have been widely assessed. The major players in hypoxia are the hypoxia inducible factors (HIFs): HIF-1α, HIF-2α, HIF-3α and HIF-1β. HIFs are stabilized under low oxygen concentration, form heterodimers and enter the nucleus, inducing the expression of their target genes by activating their promoters. HIF-1α, HIF-2α and HIF-1β are expressed in almost all tissues, whereas HIF-3α is restricted to corneal epithelium and acts as a negative regulator of HIF-1α and HIF-2α (21).

HIF-1α and HIF-2α are sensitive to oxygen concentration; they form heterodimers with HIF-1β and bind to p300/CBP. The binding sites for the heterodimers are occupied with –OH molecules at high O_2_ concentration. These couplings are only possible when the oxygen concentration is low. The main regulators of the sensitivity to oxygen are the Von Hippel Lindau protein (VHL) and the Factor Inhibiting HIFs (FIH). VHL is an ubiquitin ligase that is activated by an –OH residue in two prolines of HIF-1/2α (Pro-402 and Pro-564 for HIF-1α, Pro-405 and Pro-531 for HIF-2α) (22). VHL recruits the elonginC/elongin-B/cullin-2 E3-ubiquitin-ligase complex and triggers HIF-1/2α degradation by the 26S proteasome (23). FIH is an asparagine hydroxylase, which reacts with asparagine residues in HIF-1α (Asn-803) and HIF-2α (Asn-851), inhibiting the interaction with p300, thereby repressing HIF-1/2α translational activation (24).

In addition, there are also oxygen-independent mechanisms of HIF regulation, such as the hypoxia-associated factor (HAF). HAF is an E3 ubiquitin ligase that binds HIF-1α from 296-400 amino acids and tags it with ubiquitin, activating proteasome-dependent degradation of the HIF-1α protein (25). Another reported regulator of HIF-1α is the heat shock protein 90 (Hsp90). Hsp90 binds HIF-1α, activates E3 ubiquitin ligases, and triggers HIF-1α degradation in both normoxia and in hypoxia (26). Moreover, the degradation of HIF-1/2α modulated by VHL is also sensitive to SUMOylation, which allows VHL binding to HIF-1/2α without hydroxyproline. This process can be reverted by another protein, SENP1, which induces HIF1-α stabilization (27).

**Figure 1:**
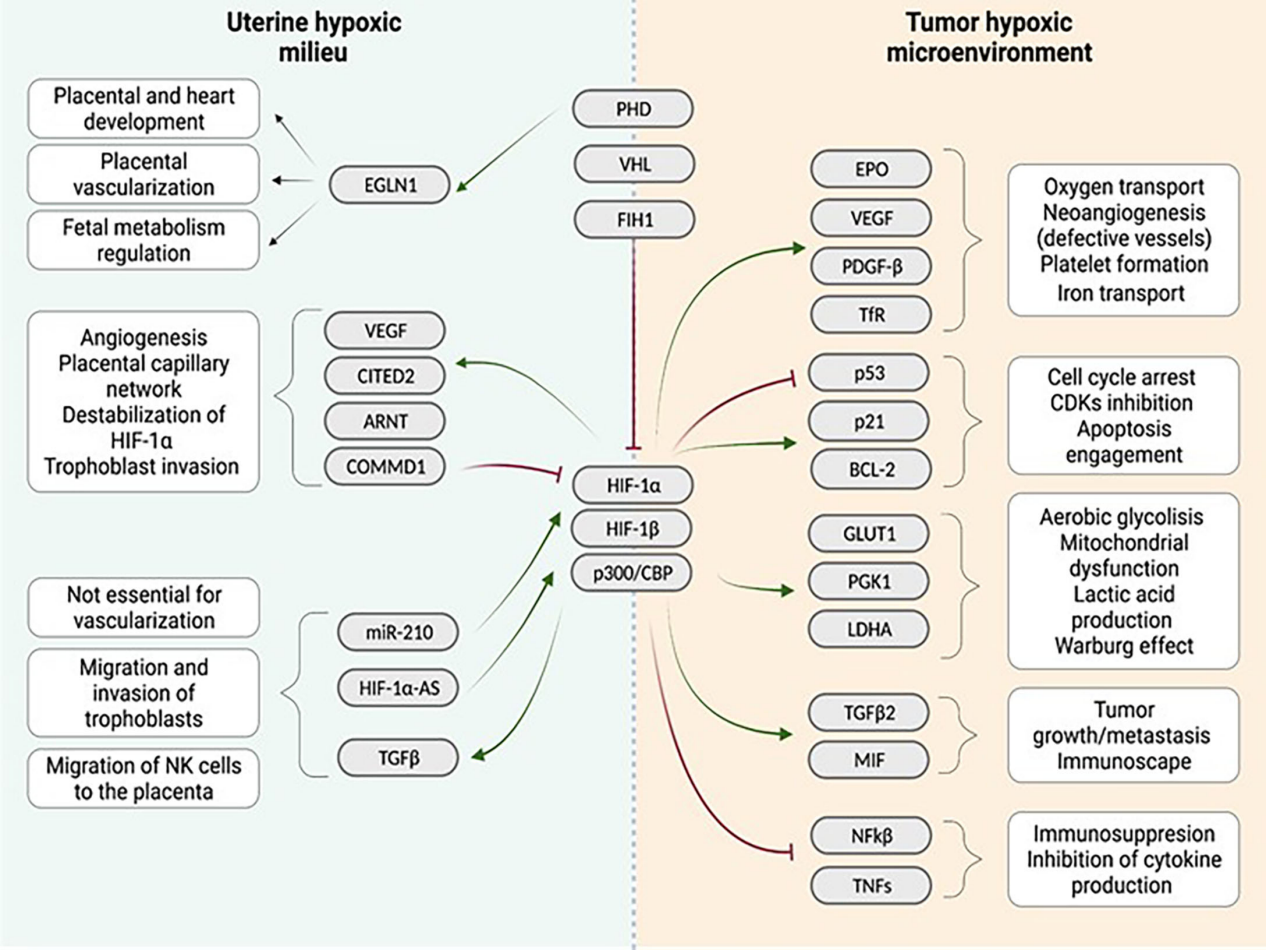
Comparison of the uterine hypoxic milieu and the tumor environment regarding the influence of the hypoxia associated gene cascade: Both niches show different interactions with a quite similar range of genes identified in the downstream cascade following hypoxia induction in the tissue. Similarities between both niches are enhanced vascularization after hypoxia induction. (Red arrows indicate inhibition, green arrows indicate activation). Created with BioRender.com.

Overall, in normoxia, HIF-1/2α factors are mostly degraded or inactive. On the contrary, in hypoxia, the inhibition of HIF-1/2α protein stabilization is not as strong as in normoxia. Therefore HIF-1/2α binds to HIF-1β and p300/CBP, translocates to the nucleus, and triggers the transcription of target genes. These genes include CDKN1A and CDKN1B (cell cycle arrest); GLUT1, PGK1 and LDHA (anaerobic metabolism, lactic acid production and mitochondrial dysfunction); EPO, VEGF, ARNT, CITED2, TDGF-β and TfR (oxygen transport, neo angiogenesis and platelet formation); and TGFB2 (tumor growth, immunosuppression and cell migration) (**Figure 1**).

### 1.4 NK Cells Function

Healthy functional circulating NK cells are sensitive to changes in oxygen concentration, which affects their phenotype and function. In this review, we will focus on their role in tumor surveillance and pregnancy, two environments, where NK cells have important functions: reacting against tumor cells and tolerating the fetus, respectively. The main function of NK cells is their ability to annihilate damaged cells without prior sensitization towards the hazard by releasing cytotoxic granules. These granules contain lytic agents like perforin and granzymes, which induce apoptosis in the targeted cells. Essential for the recognition of these hazardous cells are receptors expressed on the surface of NK cells. These receptors can be classified as activating and inhibitory. By utilizing both activating and inhibitory receptors, NK cells are regularly active towards a thread and remain inactive when encountering healthy self-cells. This balance is of importance to maintain health and counteract infection of the individual (28).

Activating receptors can be triggered by infected or transformed cells expressing stress induced signals (28, 29). Inhibiting receptors recognize infected, cancer or damaged cells, *via* the “missing-self” recognition, due to the lack of MHC class I molecules’ surface expression, thereby removing the inhibition and enabling the elimination of the target cells (30).

### 1.5 Hypoxic Tissue Environments

Hypoxia can be caused by several physiological processes and is a natural phenomenon associated with positive developmental effects in tissues. In contrast, pathological chronic hypoxia poses a challenge for cells and tissues with negative effects during disease. The prolonged exposure to hypoxic environments adapts the cellular mechanisms necessary to facilitate the maturation of cells according to their tissue purpose.

Two classical examples for chronic hypoxic tissues are the tumor microenvironment and the placenta during the first trimester of pregnancy. These tissues are prime examples for the facilitation of the oxygen-deprived milieu for growth and proliferation. In this review, we will compare the effect of hypoxia and HIF-1α in the tumor microenvironment and in pregnancy, respectively, summarized in **Figure 1**.

## 2 Tumor Microenvironment

### 2.1 Tumor Microenvironment Characteristics

The tumor microenvironment (TME) is a unique milieu, which is composed of tumor cells, stroma, infiltrating immune cells, cytokines, scarce nutrients and lack of oxygenation (31). The TME is not only supporting tumor growth, but also has a great impact on immune cells. Depending on the tumor entity, different cytokines with pleiotropic function are detected. In patients with pancreatic cancer, immune cells were in a suppressive state, which correlated with abundant TGF-β and IL-10 levels, as well as poor patient survival, although the pro-inflammatory cytokines IL-12, IL-6 and IL-18 were observed in serum (32). In a mouse breast cancer model, a population of cancer cells producing the pro-inflammatory cytokines IL-6 and IL-8 correlated with the promotion of tumor cell migration (33). Moreover, in murine models of melanoma, colon cancer and breast cancer, the cytokines IL-6 and IL-8 attracted circulating tumor cells (CTCs) and enhanced metastasis to mammary tissue (34). Cytokines also influence myeloid-derived suppressor cells (MDSCs), promoting their accumulation in the tumor niche. These immature immune cells are characterized by their ability to suppress T cell proliferation, dendritic cell maturation and NK cell activity (35). Lack of IL1-R in a murine model was correlated with less MDSCs in a mammary carcinoma, with reduced inflammation and with better survival (35). The role of MDSC differs depending on the tumor location. Whereas splenic MDSC immunosuppressed the antigen-specific function of CD8^+^ T, MDSCs in the TME inhibited tumor-infiltrating lymphocytes (TILs) antigen-specific MHC-I-restricted and general CD3/CD28 triggered activation. HIF-1α upregulation in tumor MDSCs increased arginine and NO production, which directly upregulated arginine and nitric oxide, with subsequent suppressive effects of MDSCs (36). In addition, the tumor cells displayed high proliferation rate leading to a rapid increase in tumor cell numbers. A direct consequence of this process is the shortage of nutrients. Lack of nourishment negatively influences immune cells. As previously mentioned, tumor cells take up higher amounts of glucose than healthy cells, leaving the immune cells with less nutrition and thereby impairing their function (6, 7, 37).

Tumor angiogenesis is another key factor in the hypoxic TME milieu. The vessels produced in the tumor are often disrupted, increasing tissue hypoxia by insufficient oxygen irrigation (38). Tumor cells in the hypoxic niche not only produce cytokines including IL-6, IL-10 and TGF-β, but also upregulate pro-angiogenic factors (VEGF, PDGF-β or EPO) that recruit pericytes and trigger neoangiogenesis. The translational relevance of these molecules has therapeutical relevance and are targeted to inhibit tumor growth (39). Neoangiogenesis is characterized by incomplete vascularization; the neovasculature within the TME is constantly growing, but endothelial cells are not terminally differentiated, and therefore vessels remain fenestrated (38, 40). The absence of a healthy vascularization impedes the infiltration of immune cells, as well as potentiates hypoxia and inhibits drug-delivery.

### 2.2 Hypoxic TME and Immune Cells

Many different immune cells are affected by the hypoxic TME, and the effects can be either positive or deleterious. Hypoxia alters the immune crosstalk by affecting the tumor-associated macrophages (TAMs) that potentiate immunosuppression by inhibiting immune cell recruitment, produce IL-6/IL-10 inducing PD-L1 expression on T cells, TNF-α affecting MAPK inhibitors efficacy against melanoma in human and mouse model, and increase metastasis and angiogenesis (41–43). Dendritic cells (DCs) in the hypoxic milieu show impaired migration, altered metabolism by overproduction of IDO and less antigen uptake, inhibiting the immune response (44). The anti-brain tumor activity of γδT cells is negatively affected by hypoxia, which reduces their NKG2D receptor expression and NKG2D-mediated tumor killing (45).

Given the fact that hypoxia stabilizes HIFs, HIFs are also involved in immune regulation in the hypoxic TME. For instance, in a mouse melanoma model HIF-1α^-/-^ CD8^+^ T cells show decreased expression of soluble factors including TNFα, IFNγ and granzyme B and tumor infiltration under hypoxia correlating with increased tumor growth (17). Moreover, ectopic expression of HIF-2α improved CD8+ T cell antitumor activity, revealing HIF-1α^-/-^ CD8^+^ T cells as a potential adoptive cell therapy tool overcoming the harmful hypoxic TME (46). HIF-1α was also reported to enhance murine Treg differentiation (47). Upon stimulation with IL-6, STAT3 is triggered, which directly binds to the HIF-1α promoter and enhances its transcriptional activity. HIF-1α synergized with TGF-β and supported Foxp3 transcription, as well as triggered RORγt and IL-17 production at the transcriptional level. Moreover, with a mouse glioblastoma model, HIF-1α affected cell metabolism of Treg. Tregs driven by oxidative phosphorylation (OXPHOS) metabolism escaped immunosuppression, whereas HIF-1α-triggered glycolysis induced immunosuppression and inhibited migration (48).

Helper T cells in germinal centers are also positively affected by HIFs, which is relevant for the humoral response and B cell interaction. In this report (15), it was shown that mTORC1/2 induces higher HIF-1/2α expression. Hypoxic T helper cells engaged B cells and maintained secretion of the cytokines IFNγ and IL-4 after stimulation of TCR (15). Altogether, this data suggests that HIFs play an important role in CD4^+^ T cells differentiation. This could be potentially relevant for others CD4^+^ T cells in the TME such as Th1-like cells and the immune cell crosstalk in the tumor niche (49).

In summary, the activity of HIF-1/2α in the hypoxic TME is a double-edged sword, with positive and negative effects in adoptive and innate immune system cells´ activity and crosstalk.

### 2.3 Hypoxic TME and NK Cells

NK cells are present in the TME, and they are crucial effector cells not only for cancer therapies but also for tumor prognosis. The presence of infiltrating NK cells is associated with less mortality in different cancers and in some cases correlates with a lower tumor grade (50). Gao et al. reported a tumor immune evasion strategy through TGF-β production, enhancing the conversion of tumor-associated mouse NK cells into intermediate-ILC1 (intILC1) (51). The intILC1 had an unactive phenotype, characterized by lower DNAM-1, TRAIL, CD69 surface expression and IFNγ/TNF-α production correlating with increased metastasis. Accordingly, TGF-β is known to repress NK cell metabolism and OXPHOS, and to reduce IFNγ production upon cytokine stimuli (52).

Hypoxia affects the balance of activating/inhibitory receptor surface expression and activity on NK cells, downregulating NKp44, NKp46, NKp30 and NKG2D expression and function without modifying ADCC activity (20). These receptors are not only regulated at protein level, but also transcriptionally. Moreover, hypoxic NK cells displayed lower cytokine secretion, but increased migration triggered by CXCR4 upregulation (18). This data suggests that hypoxic NK cells in the TME have reduced activating receptors, but increased tumor infiltrating potential.

In addition to the reduced activating surface receptors, hypoxic NK cells also display metabolic reprogramming. Human NK cells infiltrating liver tumors showed fragmented mitochondria upon tumor infiltration. NK cells with fragmented mitochondria displayed less OXPHOS, as well as lower oxygen consumption rate (OCR), indicating a lower ATP production. The infiltrating NK cells from patients with liver cancer were reverted by inhibiting the mTOR/drp1 pathway, suggesting a novel therapeutic approach using mTOR inhibitors for NK cells in solid tumors (53).

Besides mitochondrial reprogramming, there are more phenotypic changes involving metabolism in the hypoxic NK cells in the TME, such as the adenosine dependent CD73 pathway. Neo et al. revealed CD73 as an immune checkpoint, whose expression in tumor infiltrated NK cells is positively correlated with tumor growth in breast and sarcoma patients (54). After binding to 4-1BB on tumor cells, NK cells had an increased CD73 surface expression, which triggered STAT3 activation and TGF-β/IL-10 secretion correlating with reduced CD4^+^ T cells proliferation and IFNγ production. Although in this study there is no hypoxia exposure, HIF1-α is known to induce CD73 expression in different cells suggesting that this could also be the case in NK cells (55, 56). Therefore, hypoxic NK cells might even express increased CD73 levels triggered by HIF1-α.

HIF activity is associated with the phenotype changes in hypoxic cells. In particular, HIF-1α has been assessed to be highly relevant in hypoxic NK cells. Li et al. (57) reported the rescue of hypoxia-impaired phenotype in human NK cells used in chronic hypoxia culture. In this report, they unraveled HIF-1α/pSTAT/ERK as the signaling cascade responsible for triggering cell proliferation and activation, which was shown using chemical inhibitors for the pathway (57).

In addition, HIF-1α was reported to be a key regulator in tumor-infiltrating NK cells. In mice with a conditional HIF-1α KO in NKp46^+^ cells, tumor-infiltrating NK cells showed increased cytokine production. Using results from single cell RNA sequencing, an axis was deciphered involving the NFkB pathway and IL18R/IFNγ, which was downregulated by HIF1-α. Overall, tumor-infiltrating HIF-1α cKO NK cells displayed higher anti-tumor activity. Moreover, patients with solid tumors with higher expression of NK signature genes and the IL18/IFNγ pathway showed improved survival (58). On the other hand, another study demonstrated that HIF1α^-/-^Ncr1^iCreTg^ NK cells reduced tumor progression not by enhanced cytokine production and anti-tumor activity, but by inducing non-productive angiogenesis of the tumor. Decreased infiltration of HIF-1a cKO NK cells expressing the angiostatic soluble VEGFR-1 was observed within tumors resulting in a higher bioavailability of VEGF in the tumors. Although the primary tumor size was reduced, tumors had impaired vascularization associated with increased tumor metastasis and reduced immune cells (59). Together, these studies reveal different modes of action of HIF-1α in NK cells and identify HIF-1α as important checkpoint in NK cells with relevance for NK cell-based therapy.

### 2.4 Promising Novel Perspectives for Hypoxic TME and NK Cells

To date, there are several ongoing clinical trials with inhibitors targeting HIF-2α (PT2385) in phase I/II and preclinical studies inhibiting HIF-1α (PX-478) both in solid tumor and leukemia (60–62). Preliminary data suggest that HIF-1/2α inhibitors are clinically beneficial for patients; however, there is a concern about the tumor resistance developing to these therapies. To further understand how this resistance develops, it would be essential to assess how these drugs affect the anti-tumor immune responses.

NK cells display impaired cytotoxic activity against tumor cells in the hypoxic TME than in normoxic conditions. In contrast, ADCC remained unaffected by hypoxia (20). Solocinsky et al. proposed a model potentiating ADCC using an engineered NK92 cell line, named as high affinity NK cells (haNK). haNK cells are engineered with a high affinity CD16 (V/V 158 polymorphism) as well as endowed with IL-2 production. haNK performed better against the solid tumor cell lines EGFR^+^ in combination with Cetuximab and hypoxia exposure in comparison to NK cells isolated from healthy donors. The increased tumor killing under hypoxia might be due to low pSTAT3 as well as to the constant IL-2 presence (63). However, lack of *in vivo* models as well as the functional differences between NK92 and primary NK cells suggest the need for further investigation in pSTAT3 and IL-2 implication under hypoxia.

In the damaged skin, another pathological hypoxic niche (64), HIF-1α^-/-^ cKO mice displayed impaired wound healing and bacterial clearance, as well as less IFNγ production in comparison to *wild type* mice. Accordingly, mouse VHL^-/-^ NK cells showed the opposite phenotype to HIF-1α^-/-^ NK cells (64). In a mouse model of MCMV infection, HIF-1α was required in NK cells for metabolic adaptation and survival to the virus infection (65). In the TME context, there is a wide variety of tumor-associated bacteria (the tumor microbiome) (66). Kasper et al. reported that in colorectal tumors, the presence of anaerobic bacteria alters TME features *in vitro* (67). In addition to the bacteria presence, even if their relevance for the tumor development is a controversial subject, viruses such as Epstein-Barr-Virus (EBV) or Human Papilloma Virus (HPV) are associated to solid tumors (68). Hence, if NK cells can potentially encounter hypoxic TME, microbiome and virus infected cells, HIF-1α role could be even more intricate.

In summary, the TME is a complex milieu that changes and disrupts adaptive and innate immune cell interactions. HIF-1/2α are key players for immune cell regulation under hypoxia, with positive and negative impacts on their effector functions. Finally, NK cells show alterations in metabolism, phenotype and function under hypoxia. Taken together, HIFs might serve as promising targets for future cancer immunotherapies.

## 3 Physiological Hypoxia: Decidua Milieu and NK Cells

### 3.1 The Maternal-Fetal Interface

The oxygen concentration measurable around the fetal implantation site is comparable to the known conditions in the tumor microenvironment and therefore, in this review, we want to emphasize common features that can be used in immunological research regarding both circumstances. Peter Medawar with his question how a pregnancy can be sustained, although the fetus is a hemiallogeneic foreign body, was one of the first to describe the paradox of pregnancy (69).

The oxygen tension at the maternal-fetal interface proves to be instrumental in forming the fetus’s placenta during the early stage of pregnancy (70). With an oxygen concentration of 2-5%, the uterus also shows a hypoxic environment, less hypoxic, but similar to the TME (71). It has been reported that the oxygen tension is constantly rising in early pregnancy, at the end of the first trimester, until the maternal-fetal blood flow is fully established at week 12 (72). The extravillous trophoblasts connecting the fetal placenta with the maternal decidua are shown to be oxygen sensitive and to be degraded by oxidative stress (73). Also, the oxygen concentration during early pregnancy has a direct influence on the differentiation of fetal cytotrophoblasts, and their ability to invade the uterus and form the placenta dependent on their position within the uterine environment, which seems to include an oxygen gradient increasing towards the uterine surface (74, 75). HIF-1α is shown to be involved in the differentiation process and gene activation in placental cytotrophoblast, according to the oxygen concentration they encounter (76).

### 3.2 Role of NK Cells at the Maternal-Fetal Interface

Decidual NK cells (dNKs) comprise the majority of tissue-resident lymphocytes in the decidua. About 50-70% of the decidual lymphocytes are dNKs (77). Contrary to the circulating peripheral blood NK cells (pbNKs), the majority of dNKs display CD56^bright^ CD16^neg^ phenotype (78). Moreover, in stark contrast to the pbNKs, dNKs do not show cytotoxic behavior. Predominantly, they are engaged in the production of cytokines, growth factors and angiogenic molecules to further support the early pregnancy and promote placentation and angiogenesis at the maternal-fetal interface (78, 79).

Although dNKs do express the same activating and inhibitory receptors on their surfaces as the pbNKs, recent publications showed that engagement of some receptors displayed different effects at the maternal-fetal interface (80). Although being part of the innate immune system, especially at the maternal-fetal interface, evidence for “memory” was found (80). The ability to show adaptive behavior is not only found in dNK cells, but is a feature discovered in NK cells previously (81). It was shown that previous infection and exposure of NK cells to an antigen led to an increased reaction and IFNγ secretion upon re-challenge with the same pathogen (81). This feature has been studied in mice and was previously only hinted at in humans by epidemiological data (82). In 2004, studies investigating the influence of CMV infection on the human immune system revealed that a previous hCMV infection led to a significant increase in the detectable NKG2C^+^ population of NK and T cells in human blood donors (83). In the study of Gamliel et al. a similar correlation was seen in humans following the first pregnancy (80). It was reported that secondary pregnancies were accompanied by an expansion of the NKG2C^+^ population among dNK cells, which also showed an expansion of the peripheral blood NK cells inhibitory receptor LILRB1 bearing cells population. This population expansion was correlated by an increased cytotoxic dNK phenotype subset, secreting higher amounts of IFNγ and VEGFa upon stimulation than comparable samples from first pregnancies (80).

### 3.3 The Two Faces of Hypoxia During Pregnancy

dNK cells can be distinguished from pbNK cells by their display of surface receptors and by their “behavior”. Cerdeira et al. proposed an interesting angle to where dNK cells come from. In this proposed theory, hypoxia plays an important role in the adaptation of NK cells to the uterus and the pregnancy. They investigated the maturation of pbNK cells into dNK cells by the usage of a mixture of hypoxia, TGF-β1, and a demethylating agent, 5-aza-2′-deoxycytidine (Aza), reproducing the distinct features of dNK cells *in vitro* (84). This reaction towards the dNK phenotype would enable the cells to fulfill their immanent tasks at the maternal-fetal interface during the early weeks of pregnancy, including (1) the recognition of extravillous trophoblast invading the uterine tissue by their histocompatibility antigens *via* killer inhibitory receptors (KIRs) limiting the specifically trophoblast-derived remodeling of spinal arteries in the uterine wall, and (2) promote spinal artery development in the uterus (85). Therefore, the abundance and sensitivity of KIRs are one of the hallmarks of distinguishing dNK cells from pbNK cells.

The migration of NK cells towards the uterus is mediated by trophoblasts during the early pregnancy *via* secretion of a wide range of cytokines and chemokines associated with inflammation, like TGF-β, IL-6, CXCL8/IL-8, CXCL12/SDF1, and CCL2/MCP1 (86, 87). These factors are not only in charge of recruiting the NK cells, but also influence their function in the uterus, and mediate the interaction between immune cells and endothelial cells lining the uterus, leading to the remodeling of decidual spiral arterial walls (86).

During hypoxia, in the early pregnancy, not only HIF-1α is involved in modulation of the local gene expression to accommodate the changed physiology of the implantation site. Soares et al. list in their review several components of the hypoxia/HIFs signal transduction cascade involved in the gene expression modifications (88), including PHD2, VHL, FIH1, CITED2, COMMD1, and miR-210 (88). In order to evaluate the importance of the different factors involved in the hypoxia-induced pathway, numerous crucial genes identified in this pathway were investigated separately and their absence during pregnancy elucidated. The individual knockout of most HIFs subunits in mice was described as fatal. *Arnt*-KO mice show critical impairment of placental vascularization and overall trophoblast invasion into the decidua at E9.5 (76). In addition, *Hif1α*-deficient mice showed impairments of placentation, but on top of this, the deficiency emphasized the importance of this gene for dNK and trophoblast recruitment and their subsequent cell fate (89).

Another hypoxia regulated gene *Egln1*, was shown to be directly involved in pregnancy success, due to its lack being correlated with lethality of the progeny in uterus, and defects in heart development in surviving mice offspring produced by heterozygous gene depletion (90, 91). Deletions of *Vhl* lead to death in uterus due to the deficiency of vascularization, and hemorrhage and necrosis of the placenta in homozygous animals. Heterozygous offspring were reported to be viable (92). *Commd1* deletion is associated with fetal death after 9.5 to 10.5 days in uterus due to deficient vascularization and developmental retardation of the pups. Interestingly, the downregulation of *Commd1* expression was found to be associated with an upregulation of HIF-1α target genes, and an increased stability of HIF-1α (93). Other deletions of hypoxia-associated genes did not lead to fetal death in uterus, but to developmental impairments. Downregulation of *Cited2* leads to embryonic growth retardation due to disruptions in the capillary network within the placenta, and the succeeding difficulties in the nutrient transport from the mother (94). In addition, a knockout of *Fih1* does not seem to impact a negative pregnancy outcome in the murine model, but pups appear to be smaller and suffer from hypermetabolism (95). The miR-210 was proven to be non-essential for vascularization within the placenta, and therefore, no growth retardations have been seen in a miR-210 KO murine model (96). These evidence suggest that many genes involved in the hypoxia regulation in cells and tissues are directly associated with placentation and positive pregnancy outcome in mice.

On the one hand, HIF-1α is detrimental for the placentation, and the recruitment of dNKs and extravillous trophoblasts to the decidua during the first trimester of pregnancy (72, 97, 98) HIF-1α mRNA and protein upregulation was observed during preeclamptic pregnancy progression and is hinted to be involved in modulation of this malignancy (99–101).

HIF-1-AS2 is a long non-coding RNA (lncRNA) associated with HIF-1α expression modulation, and it was proven to inhibit PHLDA1, which is involved with apoptosis regulation, expression by binding to LSD1, a gene essential for demethylation, in fetal trophoblasts. This inhibition leads to invasion and migration of the trophoblasts, which are essential for proper placentation in pregnancy (102). However, prolonged hypoxia during pregnancy can lead to an increase of trophoblast apoptosis and suboptimal cell invasion during establishment of the early pregnancy, which ultimately can lead to the development of preeclampsia (74). The hypoxia regulation is achieved *via* FoXo3a (103). It has been shown that deregulation of the hypoxic pathway during pregnancy can lead to the development of preeclampsia. The downregulation of PHD-1, -2, and -3, which are regulators of the HIF-1α stability, is associated with preeclampsia (101). Nevertheless, not only destabilization of the HIF pathway has been observed to be disadvantageous for the pregnancy progression. Also, the potentiation of the signal by overexpressing HIF-1 proved to induce preeclampsia and intrauterine growth restrictions (IGUR) (101). This was not only shown in *in vivo* experiments performed on mice, but also in the clinics, in samples collected from human placenta (99, 100).

Preeclampsia is a pregnancy-specific state of disease, which affects 2-8% of all pregnancies, although this percentage varies across the world and demographic regions (104). This state is defined by increased blood pressure and proteinuria after the time of 20 weeks’ gestation and is still one of the leading causes of maternal mortality accompanied by other perinatal difficulties in the modern world (105). The increased blood pressure is sometimes accompanied by multi-organ perfusion problems, not limited to the placenta only (104). Despite being known and well observed in numerous cases, the predictions and treatment is still not entirely established, and there is only one effective cure to this disease - birth and removal of the placenta (104). Different severity levels of preeclampsia have been defined, but in the scope of this review, we will focus on the maternal-fetal interface in the extreme case. Although the leading causes of preeclampsia still remain elusive, disturbed placental development has been observed on a large scale, too large to be ignored as a possible correlation (106) In this regard, a multistep process has been identified that induces proper placentation, and includes NK cells that are homed to the uterus in the beginning of pregnancy (78, 107, 108). The proper connection of trophoblasts with the endometrial inner lining of the uterus, the decidua, is essential to enable proper perfusion of the developing placenta during the first trimester and preserve the proper oxygen limited conditions to maintain the placentation (72, 109). Jauniaux et al. reported that hypoxic conditions in the murine placenta during the first trimester only switched to normoxia once the perfusion of the placenta is completely established at the end of the first trimester (73). This time marks a stage of fast development of the decidua, since the differentiation of cytotrophoblast is driven by oxygen exposure (76). Cytotrophoblasts generated by the fetal mass proliferate in low concentration of O_2_, keep their undifferentiated characteristics, and differentiate into tissue with increasing O_2_ concentration towards the uterine wall (76). In the non-pathogenic state, this differentiation leads to the formation of the placenta, after invasion of the trophoblasts into the uterine wall (76). Insufficient connection and irregular blood flow can lead to fetal deficiencies, including restricted growth and preeclampsia (109). Developmental difficulties during this stage induces an insufficient invasion of spiral arteries in the placenta resulting in nutrition deficiencies of the fetus and irregular perfusion of the placenta, leading to hypoxic conditions at the site of invasion. These hypoxic conditions can enhance the formation of reactive oxygen species, therefore causing oxidative stress (110, 111). The reasons for limited trophoblast invasion are still not entirely discovered, but the maternal immune reaction towards the fetus in this dire situation could not be too far-fetched (112–114).

Intrauterine growth restriction (IUGR) is not a disease itself, but rather a manifestation of different states of disease, maternal and fetal, under one term. The reasons for growth restrictions can be manifold, from chromosomal aberrations of the fetus to maternal environmental and risk factors. IUGR is defined as a fetus weighing less than the 10^th^ local percentile at birth, although the birth weight varies with demographic and geographic regions, and this has to be considered in the weight assessment (115). Abnormal placental development might also be involved in the reduced weight of infants affected by IUGR (116). In addition, hypoxic disorders of the mothers can be a reason for IUGR (115).

This compendium of hypoxia-related effects in the placenta and at the maternal-fetal interface stresses the fine balance of oxygen concentration in this environment. On the one hand, hypoxia is urgently needed during the first trimester of pregnancy to home pbNK cells towards the uterus, and presumably, transform them into dNK cells to enable proper angiogenesis of the placenta and fetus. On the other hand, surpassing the appropriate amount of hypoxia will lead to a severe state of disease endangering both mother and child, as well leading to severe growth restrictions of the developing fetus. Altogether, the effects of hypoxia and its related genes are manifold during pregnancy, and mouse models show that many of the genes are actually essential for the positive outcome of pregnancy.

## 4 Conclusion

**Figure 2:**
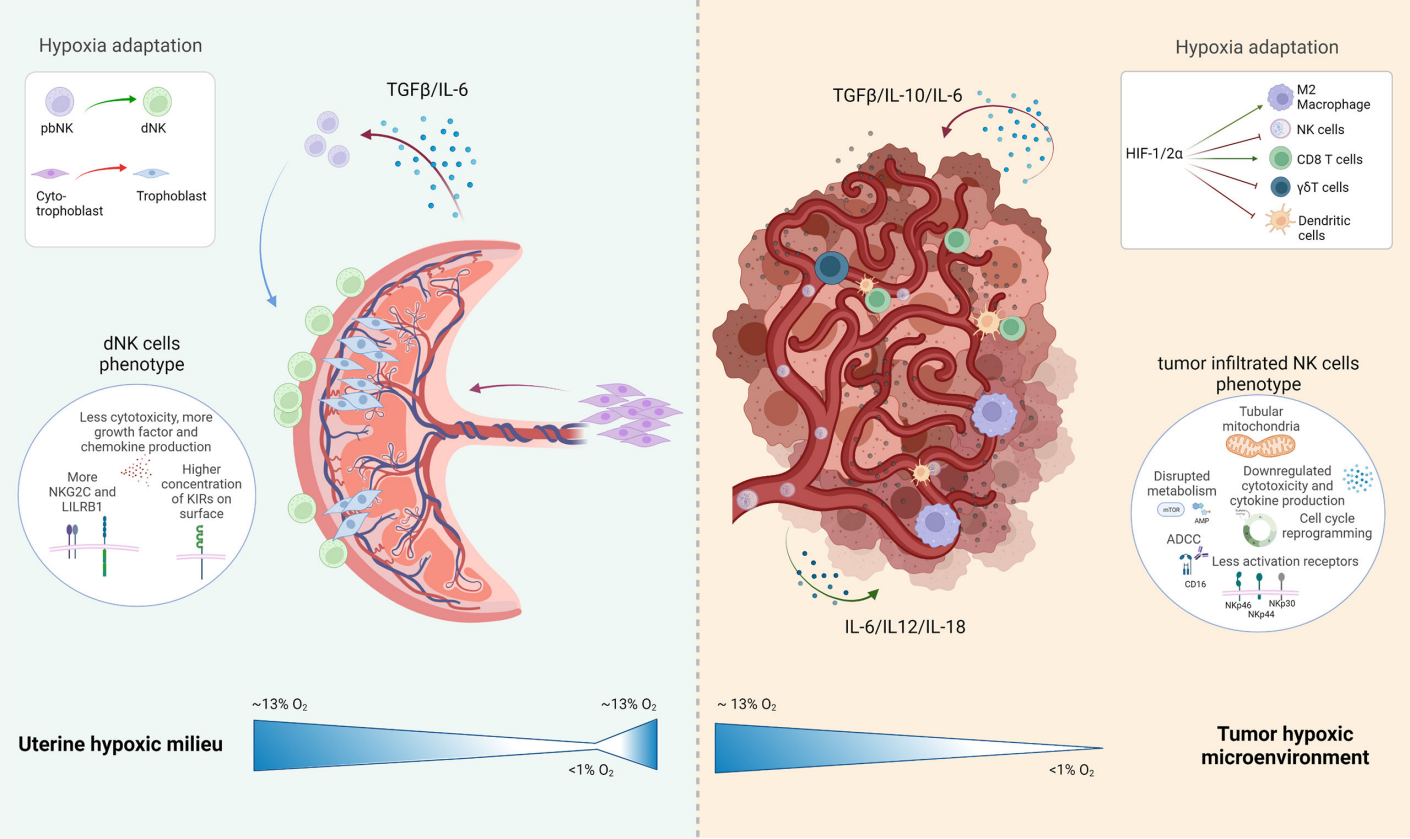
Schematic representation of the uterine hypoxic milieu and the tumor environment with special attention drawn to the present NK cell subtype and their development under hypoxic conditions: The uterine milieu shows a maturation of peripheral blood (pb)NK cells to decidual (d)NK cells, which are expressing a more angiogenic phenotype with various inhibitory NK cells receptors expressed in their surface; the tumor microenvironment depicts several inhibitory or activating effects with induced hypoxia on resident immune cells, which leads to an overall negative influence on the immune reaction at the tumor side. Created with BioRender.com.

Placenta thrives under chronic hypoxia and is even developmentally dependent on this condition. In both milieus, the placenta and the tumor microenvironment there is a high complexity and delicate balance between healthy and disrupted immune regulation. In **Figure 2**, we depicted the placenta and the tumor microenvironment and their delicate cellular interplay. As discussed in this review, the chronic hypoxic phenotype is not an immediate event, but can be rather part of a developmental and growth process as seen during pregnancy, where there is a profound impact on transcriptome and protein expression in all cells exposed in order to achieve adaptation to low oxygen concentration.

Vascularization is a crucial point in both systems. In pregnancy the oxygen gradient is essential, to provide oxygen and nutrients to the developing fetus, and facilitate correct trophoblast migration and maturation. In contrast, neoangiogenesis in the TME is unhealthy and prompted to have irregular irrigation in tissues, inefficient oxygen and nutrition distribution, which negatively affects the immune cells.

An intriguing fact about the lack of oxygenation that is also a physiological trace, is that NK cells have the ability of adapting and not damaging the surrounding tissue in the process. However, this is not always the case. Whereas chronic hypoxia in the tumor context is presumably deleterious for NK cells (affecting not only mRNA and protein expression but also metabolism), in the pregnancy context, moderate and perfectly timed hypoxia is needed for optimal fetus development, and the generation and specification of a subset of NK cells, the dNKs. However, hypoxia is also associated with negative clinical conditions in pregnancy, such as preeclampsia or IGUR. dNK cells exposed to hypoxia with sustained HIF-1α activity contribute to pregnancy failure endangering mother and fetus against their innate property. In the tumor context, the role of HIF-1α in hypoxic NK cells is yet controversial; however, previous reports suggest its pivotal role for tumor burden and disease progression.

In addition to the implication in disease development, the hypoxic conditions in the placenta are reversible. When the switch between first and second trimester is successfully executed, the pregnancy continues under normoxia. If it stays under hypoxia or if hypoxia is never reached, the pregnancy fails due to the lack of proper vascularization of the placenta and therefore lack of fetal support. In solid tumors, reversing to normoxic conditions would correlate with tumor removal or healthy vasculature, but multiple immune cells have an altered phenotype that is either more active or inhibited in the tumor niche, which makes the natural normoxic switch unlikely. Nevertheless, *in vitro* models using human NK cells, exposing them from normoxia with concomitant 1.5% O2 culture conditions partially reversed their immunosuppressed phenotype (50). Altogether, current data suggests the plasticity of both milieus, and the potential ability of NK cells to reprogram themselves depending on hypoxic-normoxic environmental conditions.

In this review, we focused on the impact of hypoxia and the role of the HIF-1α transcription factor, but its role must be considered in the context of the whole cascade of genes involved with adaptation to hypoxic conditions. Therefore, hypoxic milieus are highly heterogeneous regarding the expression and regulation of genes associated with hypoxia, with the central role of HIFs in vascularization and metabolism.

The ability of NK cells to enhance tumor clearance is abrogated in the hypoxic “cold” TME. Considering potential factors that could reverse the TME to a “hot” tumor status, manipulation of the hypoxia pathway as well as angiogenesis-related proteins in NK cells showed promising results *in vitro* and *in vivo* so far. However, the role of HIF-1α in NK cells in the hypoxic TME is still controversial. On the one hand, it could enhance a “hot” tumor status by producing healthy vasculature and therefore enhancing drug delivery. On the other hand, previous reports suggest the negative effect of HIF-1α for cytokine production and tumor surveillance promotion. In order to answer these questions, further research assessing the correlation between mouse lines and human NK samples should be performed. Comparing the findings in the TME so far with the gene regulation cascades found at the maternal-fetal interface regarding the dNK subset, there is strong evidence that hypoxia-related cell alteration is used during the tissue maturation in order to modify the activity of immune cells. The uterus proves to be a hypoxic environment during the first trimester of pregnancy, and we see a very distinctive switch of the formerly cytotoxic pbNK cells to the less cytotoxic and angiogenesis-supporting dNK cells through exposure of hypoxia and cytokines (84). This event can potentially show us a way in the future to manipulate cells in order to change their behavior to improve their efficacy for immunotherapy of cancer patients, by adapting them to a different milieu.

The more we learn about hypoxia, the more implications can be linked, which could be a source for therapeutic approaches surpassing the limitations of these specific environments. In this regard, hypoxic environment is found in many different tissue compartments (both in developmental processes and malignancies). Due to the improvement methods and possibilities of investigation, the field of immunology (innate and adaptive) and hypoxia, is expected only to grow in importance. For example, the field of lymphocytes in hypoxic environment its deeming its own review in topics such other lymphocyte subsets (such as ILCs) in other aspects of hypoxia. Findings from the hypoxia-field could inspire novel approaches of immune therapies that can be utilized not only in cancer treatment, but also in treatment of adverse pregnancy outcomes.

## Author Contributions

IG-L and RK drafted and conceived the manuscript and figures. AC and OM reviewed the manuscript. All authors contributed to the article and approved the submitted version.

## Funding

The project was supported by a network grant of the European Commission (H2020-MSCA-MC-ITN-765104-MATURE-NK), and the German Research Foundation: SFB1366 (Project number 394046768-SFB 1366; C02 to AC), SPP 1937 (CE 140/2-2 to AC), TRR179 (TP07 to AC), SFB-TRR156 (B10N to AC), and RTG2727 – 445549683 (B1.2 to AC) and RTG 2099 (Project number: 259332240 - RTG2099; P9 to AC) and ExU 6.1.11 (to A.C.). Furthermore, the work was funded by the Israel Innovation Authority Kamin grant 62615, the Israeli Science Foundation ISF Moked grant 442-18, the German-Israeli Foundation for Scientific Research and Development grant 1412-414.13/2017, ICRF Professorship grant Israeli Cancer Research Fund, the Israeli Science Foundation ISF China grant 2554/18, the Ministry of Science and Technology Foundation - Deutsches Krebsforschungszentrum MOST-DKFZ grant 3-14931, the Ministry of Science and Technology grant 3-14764. The funders had no role in study design, data collection and analysis, decision to publish, or preparation of the manuscript.

## Conflict of Interest

The authors declare that the research was conducted in the absence of any commercial or financial relationships that could be construed as a potential conflict of interest.

## Publisher’s Note

All claims expressed in this article are solely those of the authors and do not necessarily represent those of their affiliated organizations, or those of the publisher, the editors and the reviewers. Any product that may be evaluated in this article, or claim that may be made by its manufacturer, is not guaranteed or endorsed by the publisher.
